# Patient compliance with medications, nasal douching, smoking cessation and long-term outcomes of surgical septorhinoplasty – a prospective series of 56 cases

**DOI:** 10.1308/rcsann.2024.0081

**Published:** 2024-09-24

**Authors:** A Garrard, T Davies, N Walker, H Raja

**Affiliations:** ^1^ Arrowe Park Hospital, UK; ^2^ Countess of Chester Hospital, UK; ^3^ Warrington and Halton Teaching Hospital, UK

**Keywords:** Rhinoplasty, Compliance, Patient, Outcomes

## Abstract

**Introduction:**

Septorhinoplasty addresses both functional and cosmetic concerns with the nose and has been shown to have consistent, long-term benefits for patients. Nasal irrigation and medication such as antimicrobials are prescribed postoperatively to improve outcomes. Patient compliance with these interventions and outcomes of surgery have not been described. We aim to describe what the effects of compliance with these interventions may be in long-term follow-up.

**Methods:**

Patients undergoing septorhinoplasty were reviewed prospectively from 2015 to 2022. At time of operation, patients were prescribed medications, saline douching and given smoking cessation advice. Patients underwent rhinoplasty outcomes evaluation (ROE) preoperatively, at four weeks, and 3, 12, 24 and 36 months postoperatively. Compliance with postoperative interventions was measured at four weeks. Statistical tests were performed.

**Results:**

A total of 56 patients underwent septorhinoplasty. Preoperative ROE scores were improved significantly at all stages of postprocedure follow-up (*p*<0.0001). Multiple linear regression found no significant differences in patients who were not compliant with medications (*p*>0.40), nasal douching (*p*>0.22), both medication and nasal douching (*p*>0.40), and a positive smoking status (*p*>0.11) at four weeks. At 3- and 24-months follow-up, there were no significant differences in ROE scores between compliant patients and those who were noncompliant with medications, nasal douching or both (*p*>0.13).

**Conclusions:**

Our data represent the only series of patient-reported outcomes from septorhinoplasty patients where compliance with nasal irrigation, smoking cessation and antimicrobials is considered. Compliance with nasal irrigation, topical antimicrobials or smoking cessation did not influence postoperative ROE scores.

## Introduction

Septorhinoplasty is a multifaceted surgical intervention that combines two distinct techniques – septoplasty and rhinoplasty – with the primary goal of addressing both functional and cosmetic concerns. Whereas septoplasty targets the correction of a deviated or misaligned septum, which can hinder normal nasal breathing, rhinoplasty focuses on enhancing the external appearance of the nose and improving mental wellbeing. As one of the main outcomes of rhinoplasty is cosmetic, subjective patient questionnaires are used to quantify surgical outcomes.

Septorhinoplasty has consistent and long-lasting positive benefits for patients in terms of both disease and nondisease-specific quality-of-life measures.^1–4^ When considering septorhinoplasty and septoplasty individually, both show high satisfaction rates.^3,5^

Medications can be given intraoperatively and postoperatively with the intention of improving surgical outcomes such as patient satisfaction and to give the patient immediate relief from postoperative swelling and congestion. Attempts to create guidance on postoperative medications have limited confidence and, at present, there is no clear high-quality guidance on what medication groups should be prescribed postoperatively.^6^ Furthermore, evidence for the use of individual medications and regimes is limited in terms of confidence.^6^ Postoperative interventions often take the form of nasal douching/irrigation and topical antimicrobials.

Compliance with postoperative medications has not been explored thoroughly in the literature, other than demonstrating that certain patient groups may be more compliant.^7^ Patient compliance with these medications may impact outcomes measures in the short and longer term. In this case series, we aim to describe compliance with medication, nasal douching and smoking cessation advice and to investigate whether compliance makes any difference to patient-reported septorhinoplasty outcomes.

## Methods

Data from patients undergoing septorhinoplasty between January 2015 and January 2022 were gathered prospectively for a single health board. Cartilaginous grafts were used in cases where septal cartilage was inadequate for reconstruction. Graft sites included pinna and heterogeneous irradiated costochondral cartilage grafts. Splints were used in all cases to limit the risk of postoperative septal haematoma. A standard perioperative analgesia management pathway was used in all cases, but compliance with this was not recorded.

At time of operation, patients were prescribed medications, saline douching and given smoking cessation advice. Table 1 displays postoperative interventions and prescriptions. Patients underwent rhinoplasty outcomes evaluation (ROE) preoperatively, at four weeks, and at 3, 12, 24 and 36 months postoperatively. Demographic information, saline irrigation, medication compliance and smoking cessation compliance data were collected. ROE scores at respective follow-up intervals were also collected.

**Table 1:** Postoperative medication regime

Postoperative intervention	Details
Hydrogen peroxide 3%	Topical application to incision site, twice a day
Ciprofloxacin drops	Topical application to incision site, twice a day
Bactroban ointment	Topical application at incision site, twice a day
Neilmed sinus rinse	Intranasal application, four times a day for six weeks
Smoking cessation	All patients given smoking cessation advice

Descriptive statistics and statistical analysis using *t*-tests were undertaken at each follow-up interval. Where possible, multiple linear regression analysis was used to identify any differences in ROE scores between patients with differences in compliance with medication, douching and smoking.

## Results

A total of 56 patients underwent septorhinoplasty and were included in the series. Of these 56 patients, 39 (69.6%) underwent open procedures, 17 were closed procedures and 2 (3.57%) patients underwent a revision procedure.

In all, 37 (66.1%) and 35 (62.5%) patients were compliant with medication and nasal douching, respectively; 14 (25%) patients were noncompliant with medications and nasal douching, whereas 30 (53.6%) patients were fully compliant. A total of 12 (21.4%) patients were current smokers and 2 were ex-smokers at the 4-week follow-up.

**Figure 1:**
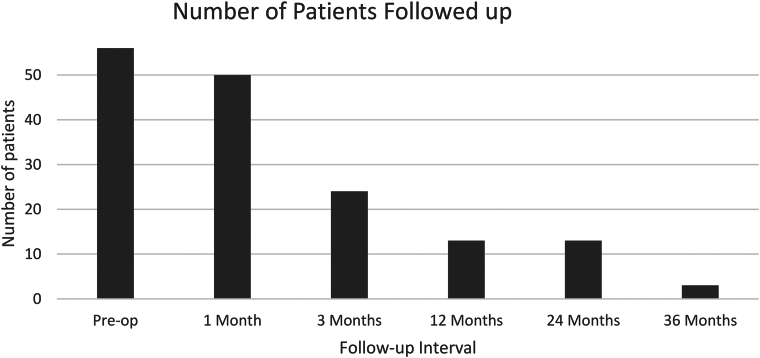
Histogram displaying number of patients followed up at each follow-up interval

The numbers of patients who submitted ROE scoring preoperatively and at four weeks and 3, 12, 24 and 36 months postoperatively were 56 (100%), 50 (89.3%), 24 (42.9%), 13 (23.2%), 13 (23.2%) and 3 (5.36%), respectively (Figure 1).

**Figure 2:**
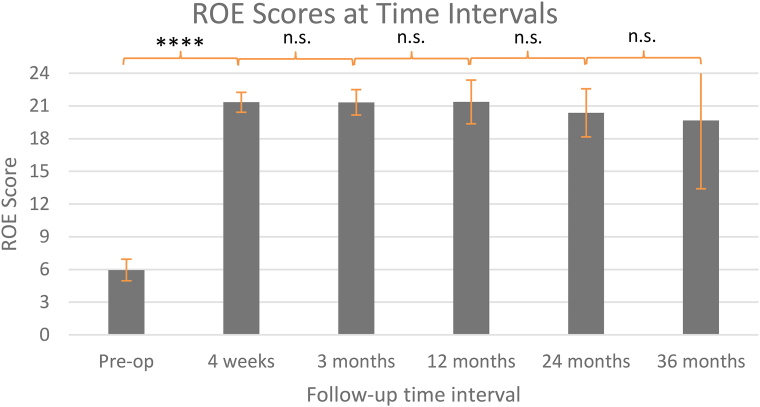
Histogram displaying ROE scores at respective time intervals. Statistically significant differences are displayed; 95% CI bars are shown. CI = confidence interval; ROE = rhinoplasty outcomes evaluation

Average ROE scores preoperatively, and at four weeks and 3, 12, 24 and 36 months postoperatively, were 5.95 (95% confidence interval (CI) 4.96–6.94), 21.3 (95% CI 20.4–22.3), 21.3 (95% CI 20.2–22.5), 21.4 (95% CI 19.4–23.4), 20.4 (95% CI 18.2–22.6) and 19.7 (95% CI 13.4–25.9) (Figure 2).

Preoperative ROE scores were improved significantly at all stages of postprocedure follow-up (*p*<0.0001), with a mean ROE improvement of 15.3 (95% CI 14.4–16.2). There was no significant difference in ROE scores between follow-up intervals (*p*>0.94).

Multiple linear regression found no significant differences in 4-week postoperative ROE scores in patients who underwent an open or closed procedure (*p*=0.28), patients who were not compliant with medications (*p*=0.40), nasal douching (*p*=0.22), both medication and nasal douching (*p*=0.40) or positive smoking status (*p*=0.11).

At 3- and 24-month follow-up, there was no significant difference in ROE scores between compliant patients and those who were noncompliant with medications, nasal douching or both (*p*>0.13) and whether they had had an open or closed procedure. There were insufficient data for multiple regression analysis at 12-month and 36-month follow-up. Result significance remained the same when Bonferroni correction was performed.

## Discussion

Our patient group represents the largest and only study looking into septorhinoplasty outcomes where compliance with saline douching, smoking cessation and topical antimicrobial medications is measured and analysed. Our ROE outcomes are in keeping with established literature showing lasting significant improvements in patient-reported outcomes.^1–5^

Our data show no improvement in ROE scores between patients who were compliant or noncompliant with postoperative smoking cessation, douching or medications (or any combination of these) at one-month follow-up. These findings were also found at 3- and 24-month follow-up when considering medication and nasal douching compliance. Smoking cessation past 1-month follow-up, and any compliance variable could not be commented on at 12 and 36 months due to insufficient data.

Our data suggest that compliance with interventions prescribed postoperatively does not impact patient-reported satisfaction and functional outcomes of septorhinoplasty in the short and longer term.

Other studies that have investigated the outcomes of septorhinoplasty considered individual postoperative interventions; these have limited outcomes and relatability for the current study due to study design and outcome measures.

In a randomised controlled, double-blinded, placebo-controlled trial, Maghsoudipour *et al* found that 3% hydrogen peroxide used postoperatively had significant improvements in clinician-assessed oedema and inflammation at eight weeks.^8^ However, this does not necessarily transfer directly to patient-reported satisfaction and functional outcomes such as ROE scoring.^9^

In our study, smoking cessation compliance did not impact ROE scoring. The effects of smoking on operative outcomes have been described with mixed and contrasting results. Studies have identified no differences between smokers and nonsmokers in patient-reported satisfaction, function, complications and revision rates with septoplasty and septorhinoplasty.^9–12^ However, in contrast, other studies have demonstrated that active smoking leads to increased rates of early complications such as perforation, slower mucociliary clearance and lower healing in septoplasty and rhinoplasty.^13–16^

Most studies do not specifically look at septorhinoplasty, and examine mostly septoplasty and/or sino-nasal surgeries in general. The only studies looking at patient-reported outcomes suggest there is no difference in outcomes.^9–11^ Comparison is therefore difficult; furthermore, there is little long-term follow-up, and outcomes measured may not correlate with longer-term patient satisfaction outcomes. Our findings agree with those studies demonstrating no difference, but smoking cessation has wide-ranging health benefits and should be advocated regardless of surgical outcomes.

The use of topical antibiotics following septorhinoplasty has not been studied in detail and therefore comparison with our outcomes is difficult. In vitro evidence suggests increases in bacterial flora in the nasal cavity postsurgery.^17^ The only study in vivo was a randomised trial by Bandauer *et al*, who found potentially reduced infectious flora in patients packed postoperatively with an antibiotic packing versus those without.^18^ None of these studies collected outcomes of postoperative infections or patient-reported outcomes, and routine postoperative packing is not recommended.^19^ Furthermore, Azizli *et al* found that ciprofloxacin was toxic to nasal epithelial cells.^6^

Systemic antibiotics in noncomplex septorhinoplasty have been found to have no benefit in decreasing infection risk in systematic review,^20^ and guidelines also adopt this approach, identifying no benefit in use beyond 24h, with no clear benefit, possible side effects and additional costs.^19^ Available data would support our findings that noncompliance with such medication would not affect outcomes.

Nasal douching or irrigation postoperatively is prescribed almost universally in sinonasal disorders given that it is inexpensive, simple, provides symptom relief, reduction of medication use and reduction of antibiotic resistance.^21^ We found no difference in ROE outcomes whether the patient was compliant or not. More patients found this hard to be compliant with than other prescriptions. Yoo *et al* evaluated compliance with saline irrigation following functional endoscopic sinus surgery and found no significant difference in sino-nasal outcome test (SNOT) scores postoperatively between compliant and noncompliant patients.^7^ Although not directly relatable due to a different sino-nasal procedure being studied, Yoo *et al* gives a useful comparison in drawing stronger conclusions.

### Limitations

Our data have some limitations that reduce the certainty of the conclusions. Compliance in this study was assessed as a yes/no outcome, and only fully compliant would satisfy compliance. There will be varying degrees of compliance, with some patients engaging wholly with treatment, some less and some not at all. Evidence is limited in assessing this impact; however, Yoo *et al* found no significant differences in SNOT outcomes in patients with differing frequencies of saline irrigation.^7^

Patient factors also may increase risk of bias. Outcomes measured in this study are patient reported and have an element of subjectivity. Acute smoking status may be underrepresented and full compliance exaggerated, especially when patients have been given advice previously in the postoperative period. ROE scoring itself is a subjective scoring system but, as one of the main outcomes of septorhinoplasty is patient mental wellbeing and cosmetic satisfaction, patient-reported outcomes may be the most relevant outcome to measure.

A significant confounder is the loss to follow-up bias, with only 5.36% inputting data at 36 months. It could be argued that this could both increase and decrease ROE score averages. Egeland *et al* found no significant differences in patient-reported nasal obstruction scores in patients responding and not responding to follow-up.^22^ Our results may be in keeping with this, as ROE scores maintained no statistical differences between follow-up periods despite follow-up rates falling. Where insufficient data were present to complete statistical analysis due to loss of follow-up, no analysis was undertaken, and no conclusions were drawn from this. Future research to overcome these limitations would include a larger patient cohort with a more complete longer-term follow-up to increase statistical certainty and allow statistical analysis at longer-term follow-up intervals. Furthermore, measuring postoperative analgesia compliance would be a variable that could be analysed.

## Conclusion

Our data represent the only series of patient-reported outcomes from septorhinoplasty patients to consider compliance with nasal irrigation, smoking cessation and antimicrobials. ROE scoring was improved significantly at all postoperative intervals compared with preoperative scores. Compliance with nasal irrigation, topical antimicrobials or smoking cessation did not influence postoperative ROE scores.
